# Chestnut Tannin/Furfuryl Alcohol Copolymers for Beech Wood Chemical Modification

**DOI:** 10.3390/polym17091159

**Published:** 2025-04-24

**Authors:** João Vitor Dorini Falavinha, Philippe Gérardin, Pedro Henrique Gonzales De Cademartori, Christine Gérardin-Charbonnier

**Affiliations:** 1 INRAE, UR 4370 USC 1445 LERMAB, Faculté des Sciences et Technologies, Université de Lorraine, 54506 Vandoeuvre-les-Nancy, France; joao-vitor.dorini-falavinha@univ-lorraine.fr (J.V.D.F.); philippe.gerardin@univ-lorraine.fr (P.G.); 2 GNanoAgro, Universidade Federal do Paraná, Curitiba 82590-300, PR, Brazil; pedroc@ufpr.br

**Keywords:** hydrolysable tannins, wood preservation, furfurylation, renewable resources

## Abstract

Tannins, present in all plants, are the most abundant polyphenols in the world. Their potential as a raw material for modifying wood alongside furfuryl alcohol (FA) has already been demonstrated in previous studies. This study focused on using large quantities of hydrolysable tannins from chestnut (*Castanea sativa*) to replace as much FA as possible to chemically modify beech wood (*Fagus sylvatica* L.). Impregnation was carried out using different concentrations and ratios of both FA and tannins and tartaric acid as catalysts through a vacuum/atmospheric pressure cycle. Copolymerization was carried out for 24 h at 120 °C. Properties such as weight percent gain (WPG), leachability, anti-swelling efficiency (ASE), thermal stability, wettability and durability against brown rot (*Coniophora puteana*) and white rot (*Coriolus versicolor*) were analyzed and compared to a furfurylation treatment without the addition of tannins. These treatments were also chemically characterized using FTIR spectroscopy. The results showed that replacing 50% of FA mass by tannins largely increased WPG and demonstrated similar leachability and dimensional stability to standard furfurylation. Above all, the new treatment showed to have better resistance to wood-degrading fungi, in addition to improved wettability and thermal stability.

## 1. Introduction

An alternative to replace the use of fossil-based materials is wood, which is considered easy-to-work, has a great strength-to-weight ratio, is renewable, naturally abundant and sustainable [1,2]. But due to its susceptibility to several micro-organisms and its dimensional instability when in contact with moisture, various attempts have been made to improve these properties of wood since the 1930s [3].

To date, most preservative treatments have involved impregnating wood with biocides, often of petrochemical origin. However, it should be noted that many products have been banned or restricted in recent years in Europe, North America and in other developed countries, due to their toxicity and fossil origin [4]. Hence, there is an urgent need for new, non-toxic, non-biocidal and sustainable preservative treatments [4]. To address these concerns, wood modification technologies have been extensively studied, with more than seven thousand articles published on the subject since 2000, and techniques such as thermal modification, acetylation and furfurylation have already been applied in industries, with a total production around 600,000 m^3^ per year [5].

Tannins, present in all plants, are the most abundant polyphenols in the world [6], and have potential as a wood preservative agent [7]. These biomolecules can be extracted using only water or with the addition of other solvents such as acetone, ethanol, methanol, sodium hydroxide and deep eutectic solvents [6,8,9]. In general, tannins are commonly classified into two large groups: condensed tannins, which are polymers based on catechin monomers, and hydrolysable tannins, which have a central sugar connected by ester bonds to galloyl groups that in turn can be connected to each other, forming complex structures [8,10].

Tannins have been used and studied for many different applications such as adhesives [11], foams for thermal insulation [12], composites [13], medicals [14], food [15], beverages [16] and water treatment [17]. In addition, these bio-polyphenols have been explored in the field of wood preservatives as biopesticides. However, due to wood’s low reactivity [18] and the high-water solubility of tannins, i.e., they easily leach out from wood after impregnation [19], necessitating cross-linkers, additives or other reactive chemical products for fixation into the wood structure. Products such as hexamine [20], boric acid [21], furfural, maleic anhydride, glyoxal, formaldehyde [22], lactic acid [23] and furfuryl alcohol [18,22] can be mentioned as examples that have already been investigated.

The potential of condensed tannins as a non-toxic and renewable raw material for modifying wood, together with furfuryl alcohol (FA), has already been demonstrated in previous studies [18,22]. FA is a chemical compound that is yellowish when pure. It can be synthesized by hydrogenating furfural, which is derived from the dehydration of xylans. Xylans, in turn, can be produced from agricultural wastes through acid hydrolysis [24]. However, in these studies, tannins and/or FA were used in low concentrations, and none of them considered the use of hydrolysable tannins.

This study focused on the use of large quantities of chestnut tannins (*Castanea sativa*), mixed with FA to form a copolymer with the aims to replace as much FA as possible with biomass that can be obtained directly through extraction.

## 2. Materials and Methods

Commercial hydrolysable tannins (THs) extracted from chestnut were supplied by King Tree (Labruguière, France). Furfuryl alcohol, purity ≥ 97% (FA), and tartaric acid, purity ≥ 99.5% (TA), were purchased from Sigma-Aldrich (Saint Quentin Fallavier, France). All the chemicals were used without any further purification before utilization.

### 2.1. Sample Preparation

Samples of European beech (*Fagus sylvatica*) with a dry density of approximately 602 kg/m^3^ were cut into three different sizes. The first group was shaped into cubes of 20 × 20 × 20 mm^3^, which were used for ASE (anti-swelling efficiency) analysis. The second group with dimensions of 25 × 15 × 5 mm^3^ was used for contact angle, decay durability tests and leaching resistance analysis. The third group, measuring 25 × 10 × 5 mm^3^, was used for termite degradation tests. Samples of modified and unmodified beech were ground for FTIR and thermogravimetric analysis.

### 2.2. Preparation of Polymeric Solutions

Wood samples from all groups were dried at 103 ± 2 °C for 48 h and weighed (m_0_) before impregnation of a solution into the wood, followed by polymerization to form a composite composed of wood and a tannin-based furan polymer. The method used was vacuum impregnation, in accordance with standard NF EN 113-1 [25], in which the samples were immersed in their respective solutions throughout the process. A single vacuum cycle was carried out at 20 mbar for 30 min, followed by a cycle at atmospheric pressure for 1 h. At the end of the impregnation process, all the samples were removed and the excess impregnation solution on the samples was carefully wiped off with a paper towel. After that, the samples were cured at 120 ± 2 °C (24 h) and stabilized at 103 ± 2 °C (24 h). Finally, each cured sample was measured again for its weight (m_1_). During the above wood modification, the weight percentage gain (WPG) was calculated as follows (Equation (1)):

WPG = [(m_1_ − m_0_)/m_0_] × 100 (1)

The beech samples were impregnated with polymerization solutions based on hydrolysable tannin and furfuryl alcohol, tartaric acid (TA) and water. These solutions were prepared with a dry concentration of 50% or 25% organic matter containing 5% of tartaric acid as a catalyst and various ratios of hydrolysable tannin (TH)/furfuryl alcohol (FA). NT corresponded to the non-treated sample.

The formulations of the treatments carried out are shown in Table 1.

### 2.3. Leachability

Leaching tests were carried out in accordance with standard NF X 41-568 [26]. Wood samples measuring 25 × 15 × 5 mm^3^ were submerged in water in different containers separated according to the type of treatment (100 mL of water was added to each sample). The containers with the water and the samples were stirred continuously throughout the process. Six leaching cycles were carried out (lasting 1, 2, 4, 8, 16 and 48 h, respectively), in which the water was replaced at the end of each cycle. Between the 8 and 16 h periods, the wood samples were kept without water for 16 h and then the process continued. At the end of all the cycles, each wood sample was dried at 103 ± 2 °C for 48 h and then weighed (m_2_). It was then possible to calculate the weight percentage loss (WPL) and the final weight percentage gain (FWPG) using the following formulas (Equations (2) and (3)):

WPL = [(m_1_ − m_2_)/m_1_] × 100 (2)

FWPG = [(m_2_ − m_0_)/m_0_] × 100 (3)

### 2.4. Dimensional Stability

To study the dimensional stability of the treated wood, the method described by Rowell and Ellis [27] was used to calculate the anti-swelling efficiency (ASE). Four replicas of 20 × 20 × 20 mm^3^ wood cubes, treated and untreated, were dried for 48 h at 103 ± 2 °C, and then weighed and measured in radial, longitudinal and tangential directions to obtain the dry volume. The cubes were submerged in distilled water for 24 h to measure their wet volume. This process was repeated three times, measuring the volume and mass at each stage. The volumetric swelling of treated and untreated wood was calculated using the following formula (Equation (4)):

S = [(V_W_ − V_D_)/V_D_] × 100, (4)

where S is the swelling of treated or untreated wood, V_W_ is the wet volume of the sample, and V_D_ is the anhydrous volume of the specimen. Volumetric swelling was calculated for each type of treated wood and untreated wood. From the swelling difference between treated and control specimens, the ASE was calculated according to the following formula (Equation (5)):

ASE = [(S_U_ − S_T_)/S_U_] × 100, (5)

where S_U_ is the volumetric swelling of untreated wood at cycle 1 and S_T_ is volumetric swelling of treated wood.

The residual mass (RM) after each cycle was calculated using the formula below (Equation (6)):

RM = [(m_c0_ − m_cn_)/m_cn_] × 100, (6)

where m_c0_ is the dry sample mass after impregnation and m_cn_ is the dry mass of the sample in cycle n, where n can be expressed from 1 to 3, representing each cycle completed.

### 2.5. FTIR Analysis

Infrared chromatography (FTIR) was performed using the Frontier IR chromatograph from Perkin Elmer (Waltham, MA, USA) to study the functional groups of each modified and unmodified beech wood, with a range of 650–4000 cm^−1^ and with 20 scans at a precision of 4 cm^−1^.

### 2.6. Thermal Stability

The modified and unmodified beech samples were mechanically ground to a fine, homogeneous powder. Then, 10 ± 1 mg of each sample was added to the Mettler Toledo model DSC 1 (Columbus, OH, USA) differential gravimetric analyzer and the STARe system to carry out the test. The test was carried out between 30 and 600 °C with a temperature increase rate of 10 °C/min.

### 2.7. Surface Properties

The wettability of the samples was determined using the contact angle, which was measured by the optical method using a Krüss (Hamburg, Germany) model DSA100 goniometer at room temperature and humidity. Water and diiodomethane were used as solvents, and the method of Owens–Wendt–Rabel and Kaelble model (OWRK) [28,29] was used to calculate surface free energy. The measurement began immediately after a drop of solvent touched the surface of the sample, after which 20 measurements were taken over the course of a minute. Each sample was subjected to three drops of each solvent. With the results, it was possible to estimate the surface energy.

### 2.8. Wood Durability

The durability test against fungi was carried out in accordance with the standard NF EN 113-1 [25]. The aim was to expose treated and untreated wood samples to wood-decomposing fungi and then check the effectiveness of the treatment by the loss of mass of the samples.

*Trametes versicolor* (white rot, TV) and *Coniophora puteanea* (brown rot, CP) were inoculated onto malt-agar culture medium in 8.5 cm diameter Petri dishes. The rots were cultivated in an incubator at a temperature of 22 ± 2 °C and a relative humidity of 70 ± 5% until their mycelium completely covered the surface of the Petri dishes. Three culture media were prepared for each treatment, and each one contained three wood samples. Of the three culture media, one consisted only of treated samples and the other two contained one untreated and two treated samples. Three culture media were also prepared for each type of rot, with only untreated beech samples as a control. For this test, both leached and unleached samples were used.

After 12 weeks, the samples were removed from the medium and gently cleaned with a brush to remove mycelium, and then dried in an oven for 48 h at 103 ± 2 °C. The following equation (Equation (7)) calculates the degradation after 12 weeks (WLD) of each sample:

WLD (%) = [(m_2_ − m_3_)/m_3_] × 100, (7)

where m_3_ is the final dry mass of the samples at the end of the test and m_2_ is the dry mass of the samples after leaching. For samples that have not undergone the leaching process and for the control samples, their dry mass after impregnation (m_1_) and initial dry mass (m_0_) have been used, respectively, instead of the dry mass after leaching (m_2_).

Durability class was determined in accordance with the EN 350-1 standard [30], which is commonly used to classify the natural durability of wood species. Samples were categorized from very durable to not durable based on the X value, which was calculated as follows (Equation (8)):

X = WLD_C_/WLD (8)

where WLD is the degradation of treated beech wood samples after leaching and WLD_C_ is the degradation of non-treated beech wood samples.

### 2.9. Statistical Analysis

The database of tests performed was studied using one-way ANOVA analysis with Tukey’s grouping using the Origin 2025 (10.2) program produced by OriginLab Corporation (Northampton, MA, USA). This analysis made it possible to classify the results into different groups; these groups are represented by letters and are organized in alphabetical order. Results that are in the same group have a statistical difference of less than 5% and can be considered statistically equal. The number of groups was not limited, so it is possible to find a different number of groups for each test.

## 3. Results

### 3.1. WPG and Leachability

Tannin-based treatments showed a WPG close to or even greater than the concentration of organic matter in the aqueous solution used for impregnation, reaching a maximum mass gain of 55%. This was not the case for samples treated solely by furfurylation, which showed a lower mass gain, achieving a maximum of 25% mass gain (Table 2) similar to results already reported for furfurylation [31]. The same behavior was observed for the treatments composed of FA and tannin, where the higher the concentration of tannin, the higher the WPG tended to be. Chestnut tannin has a much higher molecular mass and low vapor tension compared to FA, which may explain this difference in mass gain, with a part of FA being lost during polymerization.

The aim of the leachability process is to simulate outdoor conditions, where samples spend long periods in contact with water. The initial and final dry masses are then compared, to calculate the mass loss during the test. If the impregnated product is chemically bound to the wood (with covalent bonds or by intermolecular forces, such as hydrogen bonds) or simply immobilized after polymerization into the wood structure, a non-significant mass loss is expected, but if the impregnated product is just deposited in the wood, a significant mass loss is likely.

Treatment (25)TH, which did not contain FA, showed the highest mass loss, with over 50% of mass gained. And for all treatments, it was observed that the higher the concentration of the aqueous formulation, the lower the WPL. Furfurylated samples showed the lowest leaching rate and were in the same group (A) according to Tukey’s grouping. Group B, with the second lowest mass loss, was composed of treatments (50)TH(1)FA(1) and (50)TH(1)FA(2). Treatments (50)TH(2)FA(1) and (25)TH(2)FA(1) showed the highest mass loss among the furanic tannin treatments in their respective concentrations, due to the high percentage of tannin in their composition. There was probably not enough FA in the composition to copolymerize with the tannin, and this excess tannin was then easily removed from the wood.

Even with a higher mass loss, the treatments containing tannin and FA still had a final WPG twice as high as the WPG for FA treatments at all tested concentrations. These results indicated that tannins may have been copolymerized with the FA, leading to a copolymer, which may be fixed with wood or simply deposited into the wood structure since the WPL value was not elevated, especially for the treatments with a 50% organic concentration.

### 3.2. Dimensional Stability

The results are shown in Table 3. All the treatments studied showed an improvement in the dimensional stability of the wood. Among them, treatments (50)TH(2)FA(1) and (25)TH containing mostly tannin showed the lowest ASE in the third cycle, with treatment (50)TH(2)FA(1) having the most affected ASE value after all cycles. Treatments (50)TH(2)FA(1) and (50)TH(1)FA(1) showed statistically the same dimensional stability as treatment (50)FA in the first cycle. However, after three cycles, the ASE value drops drastically, and only the (50)TH(1)FA(2) treatment is statistically comparable with the conventional furfurylation treatment, since it is in the intermediate AB group. Compared to the literature [18,31], treatment (50)FA had a lower ASE than expected. This is mainly because in this study, the impregnation process was carried out without pressure cycles, which reduces the retention of FA and in turn negatively affected its ASE.

**Figure 1:**
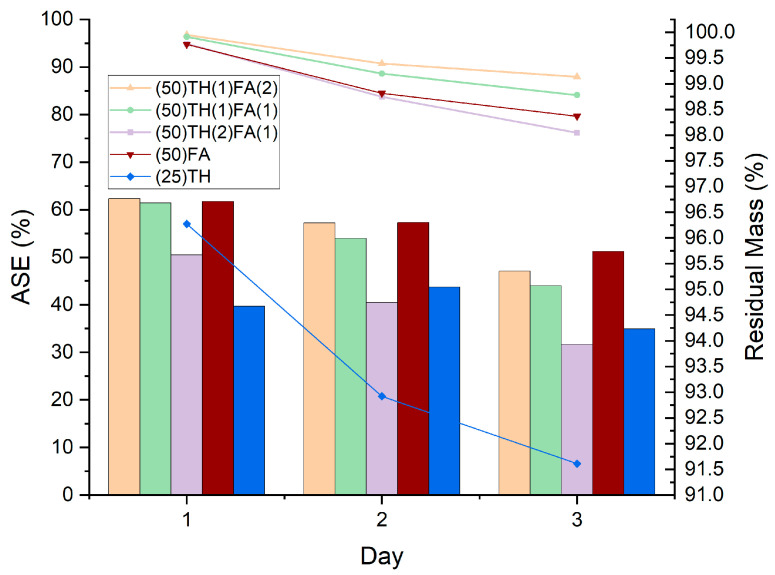
Evolution of Anti-swelling efficiency (ASE) and percentage of remaining mass (RM), where ASE is represented by columns and RM is represented by colored lines on the graph.

Figure 1 shows the evolution of dimensional stability (the colored lines on the graph) with the percentage of remaining mass compared to the mass after impregnation (columns of the graph) during each cycle. After leaching, all treatments presented lower dimensional stability in relation to the increase in mass loss. However, it can be noticed that, even with a much higher mass loss due to leaching, treatment (25)TH has an ASE value statically equivalent to the treatment (50)TH(2)FA(1).

### 3.3. FTIR Analysis

**Figure 2:**
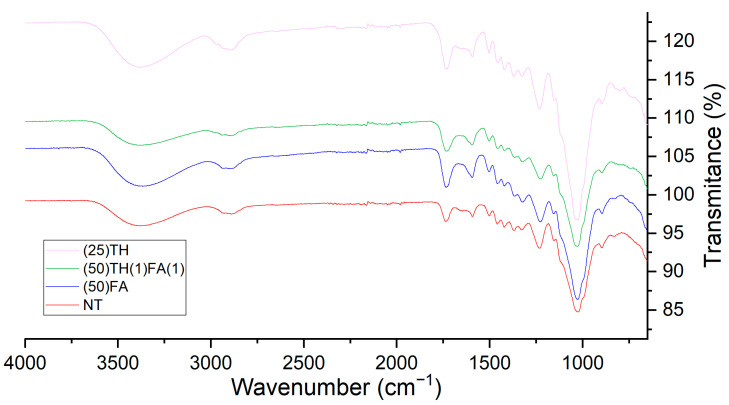
FTIR spectra comparison of samples treated only with FA, only with TH, with the combination of TH and FA and untreated sample.

Figure 2 shows the FTIR spectra of the different impregnated samples. All the treatments exhibited a very similar spectrum.

When comparing untreated wood spectrums with conventional furfurylation treatment, we can see two main differences. The first peak near 1660 cm^−1^ appears broader when compared to the untreated wood, which indicates the presence of a greater number of C=C bonds present in FA and the second peak is at 830 cm^−1^, which represents the C-O bond also present in FA.

When comparing the untreated wood with the treatment using only tannin, two differences were observed: the first in the 1595 cm^−1^ peak, which is more predominant and broader in the wood treated with chestnut tannin than in the untreated wood. This peak indicates the presence of aromatic rings, which is abundant in chestnut tannin due to the presence of galloyl groups in its structure. The second difference is in the 1330 cm^−1^ peak, which in untreated wood has the same size as its neighboring peak at 1370 cm^−1^, but in the case of treated wood, this peak is significantly larger than its neighboring peak. This indicates a greater presence of the C-O bond, which is also present in the galloyl groups.

When comparing the new treatment that mixes tannin and furfuryl alcohol with untreated wood, we observed all differences described above for the (50)FA and (25)TH treatments at the time. This is because it uses the same proportion of furfuryl alcohol and tannin to modify the wood.

### 3.4. Thermal Stability

**Figure 3:**
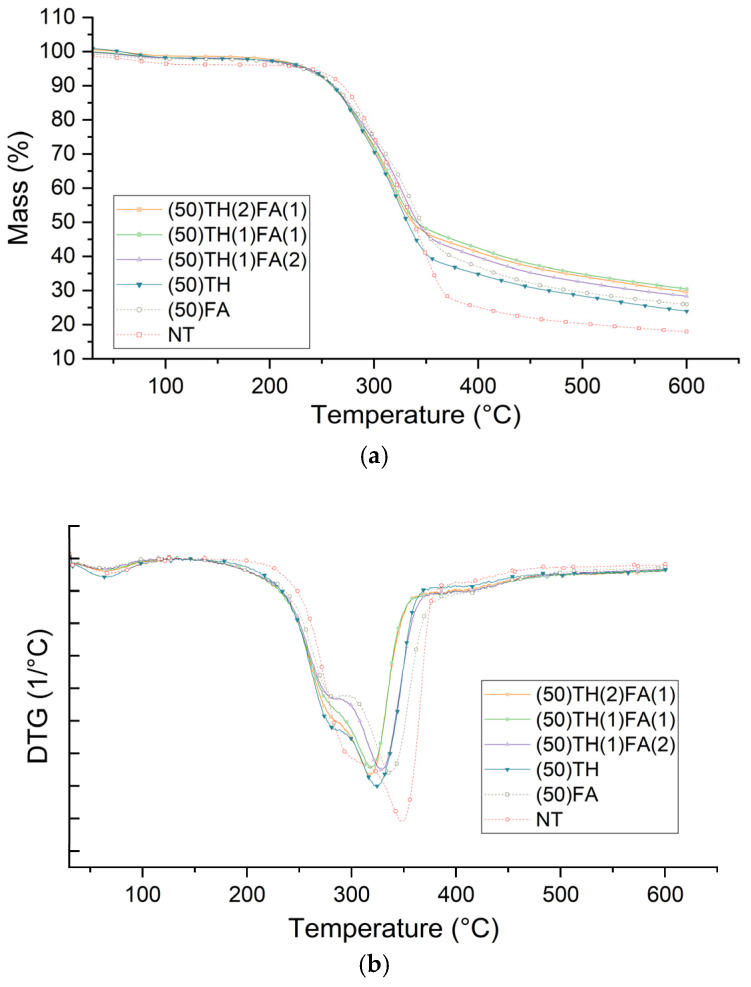
Thermal stability analysis of all treatments studied: (**a**) TG curves and (**b**) DTG curves.

Figure 3 shows thermal stability of impregnated samples.

TGA curves showed an improvement in thermal stability for all treated samples characterized by a lower weight loss up to 600 °C compared to untreated wood. Tannins themselves are known to have flame-retardant properties and to be highly stable at high temperatures [32]. Moreover, polyfurfurylalcohol polymers are also known as thermostable biobased resins [33]. These characteristics may explain the higher percentage of remaining mass in the tannin and FA treatments compared to the control. Mixed treatments associating HT and FA lead to higher char contents at the end of the TGA. The (50)TH(1)FA(1) treatment had the highest percentage of remaining mass, with 30.5% of the original mass left after the process, a gain of 5 percentage points compared to the (50)FA treatment and 12.5% of untreated wood (NT). The treatment with hydrolysable tannin alone (TH) showed a similar behavior in the wood’s thermal stability comparatively to (50)FA treatment even though its weight percent gain after impregnation and curing was lower.

By analyzing the DTG curves (Figure 3), we can identify the first thermal degradation interval between 30 and 100 °C for all the samples, which represents the evaporation of the remaining moisture present in the samples. The second stage of degradation began around 155 °C for most of the samples, except for the NT sample, which started at around 180 °C. This degradation stage lasts until 480 °C, but it is possible to observe three distinct phases. The first phase shows an accelerated loss of mass that occurs up to around 300 °C. This decomposition is attributed in part to the degradation of hydrolysable tannins reported in the literature to occur under 300 °C [34] for HT samples, but also to the chain scission of some weaker bonds (e.g., ester bonds) formed during the polymerization reaction of furfuryl alcohol catalyzed by tartaric acid [35]. The second phase takes place until 380 °C, corresponding to wood cell wall polymer degradation. The maximum of the degradation peak changes for each treatment, with the highest temperature peak occurring in the NT treatment at 350 °C and the lowest in the (50)TH(1)FA(1) treatment at 320 °C, indicating a higher susceptibility to the degradation of modified samples, even if their carbon content at the end of the TGA is higher comparatively to control. This phase is related to the degradation of hemicelluloses, lignin and crystalline part of cellulose, but also FA polymers [35,36,37,38]. Furfuryl alcohol exhibits a strong chemical affinity with lignin. This high affinity explains why furfurylation affects the thermal stability of the wood primarily at high temperatures, as lignin begins to thermally decompose only at elevated temperatures [38]. The third phase continues up to 480 °C, with a much lower degradation rate, probably linked to the carbonization of lignin [37].

### 3.5. Surface Properties

**Figure 4:**
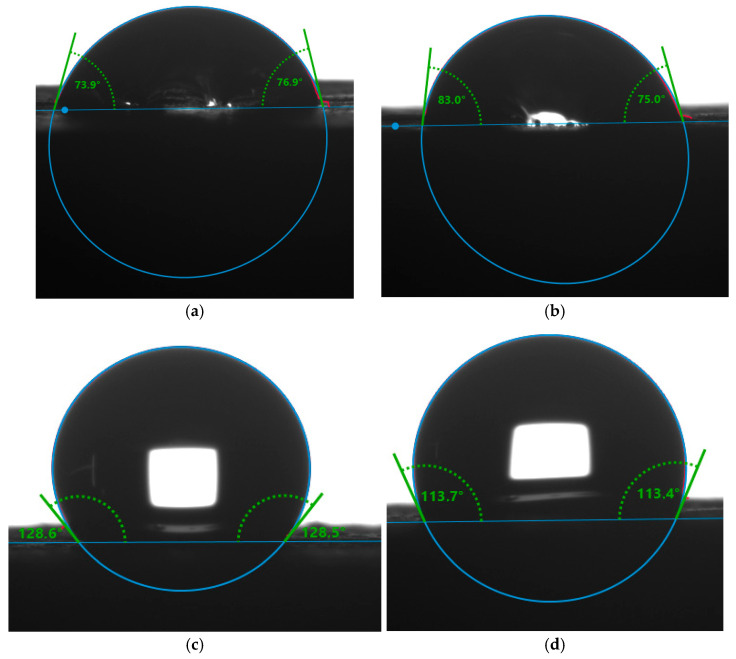
Water contact angle after 60 s for (**a**) non-treated wood (NT), (**b**) (25)T(1)FA(0) treatment, (**c**) (50)T(1)FA(1) treatment and (**d**) (50)T(0)FA(1) treatment.

Contact angles, surface free energy and behavior of water droplets on the surface of control and modified samples are reported in Table 4 and Figure 4.

A surface is considered hydrophobic if the contact angle formed with a droplet of water is higher than 90°. Furfurylated wood samples with or without tannin presented hydrophobic behavior, where the treatment (50)TH(1)FA(1) presented the highest water contact angle and higher surface energy. This energy is related to surface tension, which is the amount of energy needed to increase the surface area of a liquid. A higher surface energy means that the surface is more resistant to changes, such as the formation of droplets [39]. Treatment with (50)FA presented higher surface free energy and higher water contact angles comparatively to treatment with (25)TH, which presents less effects on the surface hydrophobicity. This behavior may be explained by the structure of HT, which presents numerous hydroxyl groups that are able to form hydrogen bonds with water.

Direct observation of this aspect of droplets of water on the surface of the different materials corroborates already described phenomena. Furfurylation treatment of wood increases its surface hydrophobicity, with or without the addition of tannin. However, treatment with tannin alone is not enough to increase this property, showing no difference from untreated wood. This behavior is clear when examining the images above (Figure 4).

**Figure 5:**
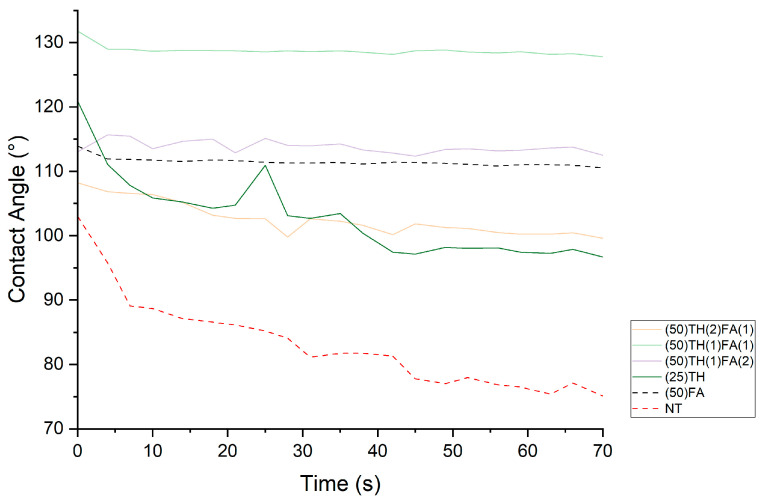
Evolution of the contact angle for 70 s of each treatment studied.

Analysis of the evolution of contact angle with time is depicted in Figure 5.

By analyzing the contact angle of each treatment over the course of 70 s, we can see that the untreated wood and the (25)TH treatment show a clear decrease in contact angle, starting at 103° and 120°, respectively, and ending at 75° and 96°. On the other hand, contact angles recorded on wood treated with FA are stable over the 70 s time course.

### 3.6. Wood Durability

The durability test against decay, white rot [*Trametes versicolor* (TV)] and brown rot [*Coniophora puteanea* (CP)] showed that almost all modified woods with or without the leaching process presented far lower mass loss values due to decay than untreated wood (Table 5). All the treatments were more effective against CP than against TV. Positive results were obtained for beech samples modified with tannin and FA, with a concentration of 50%, leading to a degradation of less than 5%. They also showed higher protection than treatment (50)FA after leaching. It should be noted that samples treated with 50% concentrated solutions and not leaching show a slightly higher mass loss than leached samples, but this is due to partial leaching in the malt-agar medium during durability tests.

**Figure 6:**
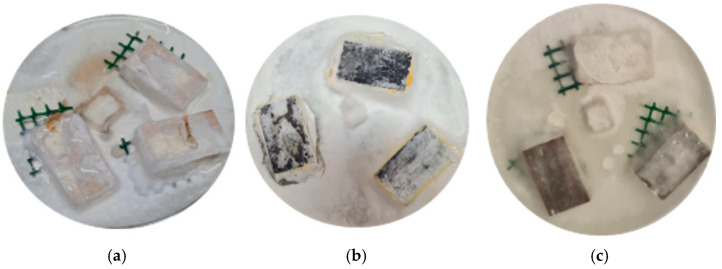
Photo of wood samples after 12 weeks exposed to TV rot: (**a**) three NT samples; (**b**) three (50)TH(1)FA(1)-treated samples; (**c**) two (50)FA-treated samples and one NT sample (completely covered by the rot).

It is worth mentioning the treatment (50)TH(2)FA(1), which uses a large amount of tannin in its composition and suffered only 2.7% degradation, showing that it is possible to replace two out of three of the FA with tannins when furfurylating wood and obtaining higher protection than the conventional furfurylation process. Another interesting result is that the treatment that used only tannin (25)TH, when it is not leached, showed considerable resistance to degradation, but after leaching, the durability class dropped to class D4. This shows the antifungal potential of tannin and that its main problem is that it is easily removed from the wood. It can be concluded that a large proportion of furfuryl alcohol can be replaced by tannins, maintaining, and in some cases even improving, the protection against fungi. All treatments studied did not prove to be biocidal, as the two rots grew on the samples during the experiment (Figure 6).

## 4. Conclusions

Wood modification using a copolymer of FA and chestnut tannin in an acidic environment with up to two-thirds of tannin instead of furfuryl alcohol showed an improvement in resistance against fungi and a more hydrophobic wood surface. The results were also equivalent to the conventional furfurylation treatment in terms of dimensional stability. Even if susceptibility to degradation is higher for modified samples, TGA indicated lower thermal degradation of samples modified with combinations of HT and FA comparatively to untreated wood. This ability to carbonize could be an important parameter for the resistance to fire. Even though there was a higher mass loss during the leachability test, it was observed that it is still possible to replace up to 50% of FA with tannin without losing its desired properties and even increasing its resistance to fungi and thermal stability. These first original tests showed that it was possible to replace furfuryl alcohol, derived from the chemical transformation of a biobased compound (furfural), with hydrolyzable tannins simply extracted from wood. This new modification of the wood opens new opportunities for further studies into new ways of protecting it with greater ecological responsibility and using other types of polyphenolic compounds. It is now necessary to complete this study by measuring the various mechanical properties, torsion resistance properties, termite resistance properties, etc.

## Figures and Tables

**Table 1:** Formulation of treatments used.

Treatment ID	Tannin Ratio	Furfuryl Alcohol Ratio
* (n)TH(2)FA(1)	2	1
* (n)TH(1)FA(1)	1	1
* (n)TH(1)FA(2)	1	2
* (n)FA	0	1
* (n)TH	1	0

* n represents the organic concentration of the given solution; n can be equal to 50 or 25.

**Table 2:** Weight percent gain (WPG), weight percent loss (WPL) and final weight percent gain (FWPG) for each treatment studied before and after leaching.

ID	WPG (%) *	WPL (%) *	FWPG (%) *
(50)TH(2)FA(1)	55.4 ± 3.2 A **	4.9 ± 0.2 C	48.3 ± 3.5 A
(50)TH(1)FA(1)	50.5 ± 2.6 B	4.0 ± 0.4 B	45.2 ± 2.9 B
(50)TH(1)FA(2)	49.1 ± 2.7 B	3.8 ± 0.3 B	42.9 ± 1.9 B
(50)FA	25.2 ± 2.0 CD	1.5 ± 0.2 A	23.8 ± 2.2 C
(25)TH(2)FA(1)	26.6 ± 1.2 C	8.2 ± 0.3 F	16.6 ± 0.9 D
(25)T(1)FA(1)	25.5 ± 1.2 CD	5.5 ± 0.6 D	18.9 ± 1.7 D
(25)TH(1)FA(2)	19.7 ± 1.1 E	6.3 ± 0.6 E	12.3 ± 1.5 E
(25)TH	24.0 ± 1.6 D	14.4 ± 0.9 G	6.0 ± 1.7 G
(25)FA	10.3 ± 0.9 F	1.6 ± 0.2 A	8.6 ± 1.2 F

* Values represent an average of at least 14 samples. ** Each letter represents one different group, results within the same group have a statistical difference of less than 5%, results with two letters have characteristics of both groups.

**Table 3:** Anti-swelling efficiency (ASE) in the first and last cycle and total mass loss for each treatment studied.

ID	ASE (%)		Total Mass Loss (%) *
	First Cycle *	Last Cycle *	
(50)TH(2)FA(1)	50.5 ± 4.7 B **	31.7 ± 2.6 C	2.0 ± 0.1
(50)TH(1)FA(1)	61.4 ± 1.0 A	44.0 ± 1.1 B	1.2 ± 0.1
(50)TH(1)FA(2)	62.3 ± 2.5 A	47.1 ± 2.5 AB	0.9 ± 0.01
(50)FA	61.7 ± 4.0 A	51.2 ± 1.7 A	1.6 ± 0.2
(25)TH	39.7 ± 5.1 C	34.9 ± 5.3 C	8.4 ± 1.1

* Values represent an average of 4 samples. ** Each letter represents one different group, results within the same group have a statistical difference of less than 5%, results with two letters have characteristics of both groups.

**Table 4:** Water and diiodomethane contact angle and surface free energy for each treatment studied.

ID	Contact Angle (°)		Surface Free Energy (mN/m)
	Water *	Diiodomethane *	
(50)TH(2)FA(1)	107.4 ± 4.7	50.5 ± 1.2	34.04 ± 4.4
(50)TH(1)FA(1)	123.9 ± 4.8	49.1 ± 3.8	37.3 ± 0.9
(50)TH(1)FA(2)	114.9 ± 5.9	49.6 ± 1.4	35.7 ± 0.04
(50)FA	111.8 ± 0.8	47.26 ± 2.6	36.3 ± 1.3
(25)TH	99.5 ± 1.3	48.9 ± 4.0	35.0 ± 2.3
NT	88.4 ± 9.1	51.4 ± 1.8	35.6 ± 3.4

* Values represent an average of at least 60 measurements.

**Table 5:** Weight loss due to rot degradation (*Trametes versicolor* and *Coniophora puteanea*) after leaching (WLD_L_), without leaching (WLD_NL_) and the durability class by EN 350 standard [30] for each treatment studied.

ID	*Trametes versicolor*			*Coniophora puteanea*		
	WLD_NL_ (%) *	WLD_L_ (%) *	DC *	WLD_NL_ (%) *	WLD_L_ (%) *	DC *
(50)TH(2)FA(1)	6.3 ± 0.5 (BC) **	2.7 ± 0.3 (A)	D1	5.6 ± 0.7 (CD)	2.4 ± 0.3 AB	D1
(50)TH(1)FA(1)	4.7 ± 0.9 (AB)	3.3 ± 1.2 (A)	D1	4.1 ± 0.2 (C)	2.0 ± 0.5 (AB)	D1
(50)TH(1)FA(2)	4.1 ± 0.7 (AB)	1.2 ± 0.3 (A)	D1	3.6 ± 0.3 (BC)	1.3 ± 0.3 (AB)	D1
(50)FA	2.8 ± 1.6 (A)	9.3 ± 4.0 (A)	D2	1.1 ± 0.4 (AB)	0.6 ± 0.3 (A)	D1
(25)TH(2)FA(1)	5.0 ± 0.5 (AB)	25.9 ± 2.5 (B)	D4	3.3 ± 0.3 (BC)	6.7 ± 3.7 (B)	D2
(25)TH(1)FA(1)	4.2 ± 0.3 (AB)	26.1 ± 2.0 (BC)	D4	3.4 ± 0.3 (BC)	2.5 ± 2.5 (AB)	D1
(25)TH(1)FA(2)	8.1 ± 3.1 (CD)	20.5 ± 7.2 (B)	D4	3.6 ± 0.4 (BC)	0.6 ± 0.8 (A)	D1
(25)TH	10.6 ± 1.2 (D)	34.8 ± 5.0 (DC)	D5	7.4 ± 2.0 (D)	15.1 ± 9.9 (C)	D4
(25)FA	23.2 ± 1.2 (E)	26.0 ± 9.3 (B)	D4	0.6 ± 0.1 (A)	4.4 ± 5.1 (A)	D1
NT	38.6 ± 3.6 (F)	38.6 ± 3.6 (D)	-	27.6 ± 5.3 (E)	27.6 ± 5.3 (D)	-

* Values represent an average of 7 samples. ** Each letter represents one different group. The results within the same group have a statistical difference of less than 5% and results with two letters have characteristics of both groups.

## Data Availability

Data and materials are contained and presented within this article. The data that support the findings of this study are available from the corresponding author, CGC, upon reasonable request.
